# Using the Objective Structured Assessment of Technical Skills (OSATS) global rating scale to evaluate the skills of surgical trainees in the operating room

**DOI:** 10.1007/s00595-012-0313-7

**Published:** 2012-09-01

**Authors:** Hiroaki Niitsu, Naoki Hirabayashi, Masanori Yoshimitsu, Takeshi Mimura, Junya Taomoto, Yoich Sugiyama, Shigeru Murakami, Shuji Saeki, Hidenori Mukaida, Wataru Takiyama

**Affiliations:** 1 Department of Surgery, Hiroshima City Asa Hospital, 2-1-1 Kabe-minami, Asakita Ward, Hiroshima, Hiroshima Japan; 2 Department of Surgery, Division Frontier Medical Science Graduate School of Biomedical Sciences, Hiroshima University, 2-3 Kasumi 1-chome, Minamiku, Hiroshima, Hiroshima Japan

**Keywords:** Education, Global rating scale, OSATS

## Abstract

**Purpose:**

The education of surgical trainees should be based on an accurate evaluation of their surgical skill levels. In our hospital, the Objective Structured Assessment of Technical Skills (OSATS) is used for this purpose. We conducted this study to demonstrate the validity and accuracy of the OSATS for assessing surgical skills in the operating room (OR) setting.

**Methods:**

Between January, 2007 and December, 2010, the OSATS global rating scale was used to assess several operations in which surgical trainees participated. We assessed ten surgical trainees who participated as the main surgeon or first assistant, and studied the correlation between their postgraduate year and their OSATS score.

**Results:**

The median score of the global rating scale for each trainee improved with each year of experience. The median scores of all trainees in postgraduate years 3, 4, and 5 were significantly different (*p* < 0.001 for both the main surgeon and first assistant roles; Kruskal–Wallis test).

**Conclusion:**

Using the OSATS global rating scale to assess the surgical skills of trainees in the OR was feasible and effective.

## Introduction

In Japan, the training curriculum for surgeons to be certified by the board of the Japan Surgical Society [1] contains minimally required surgical elements. However, the actual training programs, the skill assessment procedures, and the feedback systems for each trainee are not described in detail and vary among hospitals. Traditionally, the surgical skills of trainees are assessed by the supervisor, with feedback, in the OR [2]. In contrast to these subjective assessments, the validity and reliability of several objective methods of assessing surgical skills have been reported previously [2]. The Objective Structured Assessment of Technical Skills (OSATS) is one of these objective skill assessments, used by the University of Toronto since the 1990s [3, 4]. The OSATS is an examination using bench model simulation, which consists of two components: an operation-specific checklist and a global rating scale. Both of these methods were reported to be proportional to the maturity of surgical skills. In particular, the global rating scale is a common method of evaluation, not limited to any specific procedures, which consists of seven evaluation items scored on a 5-point scale. In other words, the global rating scale can be applied to any other skill assessment.

The OSATS can provide valuable information, but when done as an off-the-job examination, it requires a lot of effort and a budget outside that for daily medical practices. Therefore, we attempted to apply the global rating scale to assess the surgical skills of trainees during actual operations and started a training program based on this method in 2007. We describe our method of skill assessment and present data to validate its effectiveness.

## Methods

**Fig. 1:**
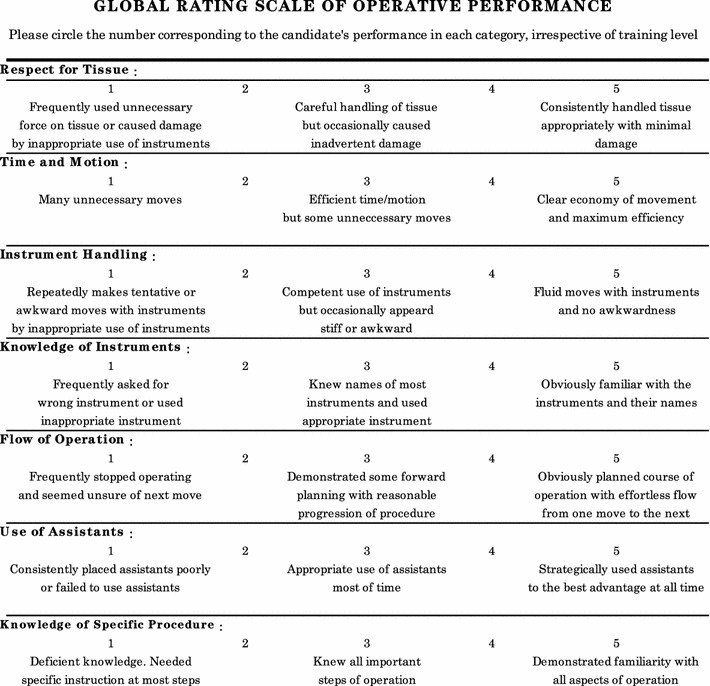
The global rating scale used in the Objective Structured Assessment of Technical Skills (OSATS) [2], which we used to score the skills of each surgical trainee in performing or assisting in real operations. Full marks are 35 points on 7 items and 30 points on 6 items, respectively, as a surgeon and as an assistant (in the case of assistant, ‘Use of Assistant’ is excluded from the scoring)

We surveyed all operations, in which postgraduate year 3, 4, and 5 surgical trainees participated as the main surgeon or as the first assistant in Hiroshima City Asa Hospital, between January, 2007 and December, 2010. The surgical skills were evaluated using the global rating scale of the OSATS (Fig. 1).

Operations were classified according to whether the trainee was acting as the surgeon or the first assistant and were based on the level of difficulty of the surgical procedure. The surgical procedures were arbitrarily classified into three groups (Table 1). The scores of each trainee evaluated with the global rating scale were collected and studied in relation to each postgraduate year.

**Table 1:** Example of the operation classification according to the degree of difficulty

Degree of difficulty	Operations
Low	Thyroidectomy
	Breast surgery
	Bullectomy, VATS
	Appendectomy (open or lap.)
	Inguinal hernioplasty
Intermediate	Open lung surgery (such as lobectomy)
	Open distal gastrectomy
	Lap. local resection of the stomach
	Open colectomy
	Lap. cholecystectomy
	Distal pancreatectomy
High	Esophagectomy
	Lobectomy of lung (VATS)
	Open or lap. total gastrectomy
	Lap. distal gastrectomy
	Lap. colectomy
	Open or lap. proctectomy
	Hepatectomy
	Pancreatoduodenectomy

Evaluations were carried out by staff surgeons who participated in the operation in a supervisory role, rather than as a third-party evaluator who watched the operation or its video, because the main purpose of our method was to educate based on feedback, rather than to simply evaluate. To ensure objectivity of the evaluation, before starting this assessment system, all evaluators watched three videos of laparoscopic cholecystectomy being performed by three different trainees, and made a standard matching of the scores.

The scores of each trainee were analyzed as the median during each of nine terms, being the first term (from April to July), second term (from August to November), and third term (from December to March) in each postgraduate year. To examine the correlation between the postgraduate year and the surgical skill evaluated by the global rating scale, the scores in the second term of each year were statistically analyzed as follows: The Kruskal–Wallis test was used to compare the three groups and the Mann–Whitney *U* test was used to compare differences between two groups. Analyses were performed using the SPSS software application and *p* values <0.05 and 0.05/3 were considered to indicate significance.

## Results

**Fig. 2:**
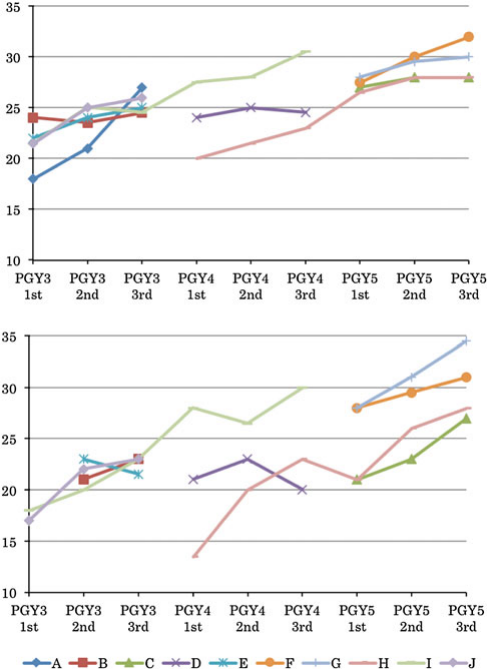
Changes in the global rating scale for each trainee as a surgeon. *Above* low difficulty, *below* intermediate difficulty

**Fig. 3:**
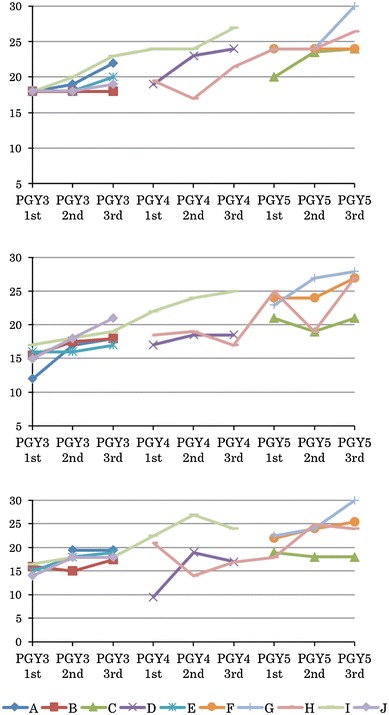
Changes in the global rating scale for each trainee as a first assistant.* Above* low difficulty,* middle* intermediate difficulty,* below* high difficulty

During the period of this study, ten surgical trainees in our hospital participated in 4240 operations; as the main surgeon in 757 and as the first assistant in 888. Their skills were assessed by the global rating scale. Figures 2 and 3 show the median scores of the global rating scale for each trainee. Figure 2 presents median scores of the operations of low and intermediate difficulty for the surgeon, and Fig. 3 presents all of the operations assessed when the trainee was acting as the first assistant. There was a tendency toward a positive correlation between the global rating scale and the postgraduate year. The same correlation was demonstrated for other periods, but we arbitrarily chose the middle 4 months to reflect our analysis.

**Fig. 4:**
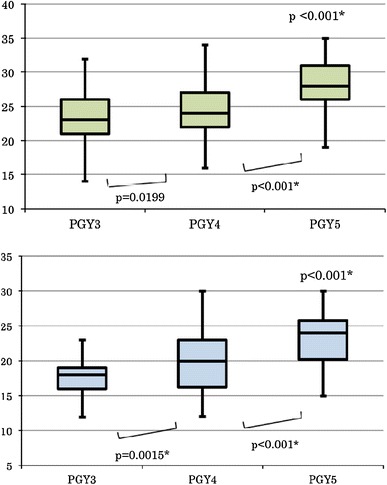
Correlation between the postgraduate year and the score. The scores were compared among the second terms of postgraduate years 3, 4, and 5. *Above* as a surgeon. *Below* as a first assistant. The differences in the scores of these three groups were significant (Kruskal–Wallis test, <0.001 and <0.001, respectively)

Figure 4 shows box-plots of the global rating scale of the trainees during the middle periods of postgraduate years 3, 4 and 5, independently for when they acted as the surgeon or as the first assistant. The scores calculated using the global rating scale increased significantly with each postgraduate year (*p* < 0.001 for both the operator and first assistant roles; Kruskal–Wallis test).

## Discussion

The Objective Structured Assessment of Technical Skills (OSATS) is a multi-station performance-based examination of surgical skills, first used by the University of Toronto in the 1990s. Stations involve bench model simulations of surgical procedures appropriate to general surgery. Eight 15-min stations are used for the examination, including the excision of a skin lesion, the insertion of a T-tube, abdominal wall closure, hand-sewn bowel anastomosis, stapled bowel anastomosis, control of inferior vena cava (IVC) hemorrhage, pyloroplasty, and tracheostomy. The examiners mark the performances using two evaluation tools: an operation-specific checklist and a global rating scale. Reznick et al. reported that both the checklist assessment and global rating scale improved with the number of postgraduate years [3].

Although this objective and off-the-job examination is useful and avoids potential harm to patients, it has not been used widely, because it requires extra staff, surgical instruments, time, and costs. Therefore, we applied this assessment to real operations performed from January, 2007 onward, easily and objectively without any special preparations. Because the global rating scale assessments are not limited to a specific procedure, our method can be applied to various kinds of surgery.

After the completion of each operation in our hospital, the supervisor evaluated the trainees’ surgical skills using the global rating scale, and entered the results into a database, sometimes with debriefing, as soon as possible. The database is easy to add to and to reference, so we think it is a useful method for providing feedback to surgical trainees. In the medium and long term, it is also easy to show the trainee their learning curve by extracting data from that database. In our hospital, the changes in scores were discussed with each trainee every 6 months, to ensure they were progressing and thus, keeping them motivated.

We demonstrated positive correlations between the postgraduate year and the scores obtained with the global rating scale. Traditionally, blood loss and operation time have been used as a measure of surgical skills; however, these values differ on a case-by-case basis, depending on the patient background or other circumstances, and cannot be used to assess the surgical skills of assistants. This study shows the effectiveness of the new OSATS method.

The limitations and problems of the OSATS method are as follows. First, differences in the scores may be caused by the assessments being carried out by different evaluators. Before we started this objective assessment, all evaluators watched the same video of an operation, which they assessed to help standardize the scoring; however, the standardization was not rechecked afterwards, for example, if an evaluator was transferred to another hospital and a new evaluator came to our hospital. Nevertheless, the evaluator’s bias seemed to have limited influence on the scores, because the operation supervisor for each trainee is different for each operation in our hospital, so various evaluators assess the surgical skills of each trainee, which reduces this bias. In this study, 12 evaluators assessed 10 surgical trainees. Second, the fact that the evaluators already knew which postgraduate year group the trainee belonged to may have biased the scores. Figures 2 and 3 show clear differences among the trainees, and indicate a positive correlation between the score and the postgraduate year at an individual level. This demonstrates that adequate estimation was done, regardless of the postgraduate year. Moreover, the bias might be critical for the certifying examination, but the main purpose of our method was education based on accurate and timely feedback, so this was not a serious problem from an educational point of view. Third, some operations were not assessed. In fact, only 40 % of operations that trainees participated in were assessed and some of the operations were not evaluated in a timely manner. Prompt evaluations are necessary for precise assessment and appropriate feedback. Our solution to this problem lay in the comprehensive database for all surgical cases, which included patient profiles, risk assessments for perioperative complications, surgical records, morbidities based on the Clavien–Dindo classification [5], pathological findings, prognosis, clinical trial registrations, interest in cases, and so on. We integrated the skill assessment into this comprehensive database 2 years ago, following which the ratio of missed skill assessments decreased remarkably. In 2011, more than 90 % of the operations were evaluated. Fourth, problems arise when one surgical trainee has much better or much poorer skills than other surgical trainees and there was a tendency for the surgical trainees with higher scores on the skill assessment to have had more experience as the surgeon, whereas those with lower scores were likely to have had more experience as an assistant. Skill assessments based on the level of difficulty, as an experiment, may be useful for stepwise education. For example, a trainee who achieved a very high score as the main surgeon of an operation with low difficulty, or as the first assistant of an operation with intermediate or high difficulty, should then be regarded as competent to be the main surgeon of an operation with intermediate difficulty. However, we have not yet made changes to the training program based on the global rating scale, because very few surgical trainees have been evaluated by this method. More data need to be accumulated and the skills of many more trainees need assessment for us to establish a training program that is appropriate for the trainee’s global rating scale. Finally, our evaluation method mainly aims to evaluate technical performance, but does not reflect the impact of non-technical skills such as decision making, communication, leadership, and so on. The reason for this is that the global rating scale of OSATS was started for bench model examination or living animal examination. However, non-technical skills are as important as technical skills and recent reports document that an assessment or scoring system for non-technical performance is useful [6–8]. Non-technical-skill evaluation could be added to our method potentially, because both the evaluation and the modified global rating scale of OSATS are premises for using in OR. Adding the non-technical-skill evaluation to our evaluation system is the next challenge for a more precise evaluation of a trainee’s performance in actual operations.

In conclusion, our skill assessment method, using the global rating scale of the OSATS in an on-the-job manner, is validated by the fact that the scores improved with advances in postgraduate years. Although this method has some problems and limitations, we believe it has a positive impact on the education of surgical trainees.

## References

[1] Japan Surgical Society. Training curriculum for Board Certified Surgeon, revision on April 1st, 2009. http://www.jssoc.or.jp/procedure/specialist/curriculum-1.html

[2] van Hove PD, Tuijthof GJ, Verdaasdonk EG, Stassen LP, Dankelman J (2010). Objective assessment of technical surgical skills. Br J Surg.

[3] Reznick R, Regehr G, MacRae H, Martin J, McCulloch W (1997). Testing technical skill via innovative “bench station” examination. Am J Surg.

[4] Martin JA, Regehr G, Reznick R (1997). Objective structured assessment of technical skill (OSATS) for surgical residents. Br J Surg.

[5] Clavien PA, Barkun J, de Oliveira ML (2009). The Clavien–Dindo classification of surgical complications: five-year experience. Ann Surg.

[6] Yule S, Flin R, Paterson-Brown S, Maran N (2006). Non-technical skills for surgeons in the operating room: a review of the literature. Surgery.

[7] Yule S, Flin R, Maran N, Rowley D, Youngson G, Paterson-Brown S (2008). Surgeons’ non-technical skills in the operating room: reliability testing of the NOTSS behavior rating system. World J Surg.

[8] Crossley J, Marriott J, Purdie H, Beard JD (2011). Prospective observation study to evaluate NOTSS (Non-Technical Skills for Surgeons) for assessing trainees’ non-technical performance in the operating theatre. Br J Surg.

